# Analysis of the Robustness of Network-Based Disease-Gene Prioritization Methods Reveals Redundancy in the Human Interactome and Functional Diversity of Disease-Genes

**DOI:** 10.1371/journal.pone.0094686

**Published:** 2014-04-14

**Authors:** Emre Guney, Baldo Oliva

**Affiliations:** 1 Center for Complex Network Research, Northeastern University, Boston, Massachusetts, United States of America; 2 Structural Bioinformatics Group (GRIB), Departament de Ciències Experimentals i de la Salut, Universitat Pompeu Fabra, Barcelona, Catalonia, Spain; Mathematical Institute, Hungary

## Abstract

Complex biological systems usually pose a trade-off between robustness and fragility where a small number of perturbations can substantially disrupt the system. Although biological systems are robust against changes in many external and internal conditions, even a single mutation can perturb the system substantially, giving rise to a pathophenotype. Recent advances in identifying and analyzing the sequential variations beneath human disorders help to comprehend a systemic view of the mechanisms underlying various disease phenotypes. Network-based disease-gene prioritization methods rank the relevance of genes in a disease under the hypothesis that genes whose proteins interact with each other tend to exhibit similar phenotypes. In this study, we have tested the robustness of several network-based disease-gene prioritization methods with respect to the perturbations of the system using various disease phenotypes from the Online Mendelian Inheritance in Man database. These perturbations have been introduced either in the protein-protein interaction network or in the set of known disease-gene associations. As the network-based disease-gene prioritization methods are based on the connectivity between known disease-gene associations, we have further used these methods to categorize the pathophenotypes with respect to the recoverability of hidden disease-genes. Our results have suggested that, in general, disease-genes are connected through multiple paths in the human interactome. Moreover, even when these paths are disturbed, network-based prioritization can reveal hidden disease-gene associations in some pathophenotypes such as breast cancer, cardiomyopathy, diabetes, leukemia, parkinson disease and obesity to a greater extend compared to the rest of the pathophenotypes tested in this study. Gene Ontology (GO) analysis highlighted the role of functional diversity for such diseases.

## Introduction

A fundamental characteristic of biological systems is tolerance to noise. The ability to counteract both internal mechanistic failures and changes in environmental conditions plays a central role in the survival of the organism. The main components of robustness are controlling the system through negative and positive feedback [1], splitting the parts of the system as functional units [2] (modularity and decoupling), and phenotypic plasticity [3] (typically achieved by redundancy). In a biological system, groups of genes are optimized in functional decoupling, redundancy and diversity such that the effects of perturbations are minimized [4]. However, complex biological systems have to balance between robustness and fragility which implies that a small number of rare perturbations can substantially disrupt the system [5]. In particular, some mutations are the main cause of diseases by exploiting the fragility of the biological system.

During the past decade, genome-wide efforts such as linkage analysis and association studies have successfully associated numerous causal loci with human disorders [6]. Still, much effort needs to be taken to fully understand the complex implications on the whole system. For this purpose, several methods have been developed recently to amplify available disease-gene associations using the principle of “guilt-by-association” through underlying biomolecular networks. These methods typically exploit relationships of the disease causing genes with other candidate genes, using the neighborhood of known associations in the physical [7], [8] or functional [9] interaction network. Recent methods extend the definition of the neighborhood to account for the global topology of the underlying network (network-based disease-gene prioritization) [10], [11]. Such global topology based methods have been showed to improve disease-gene association prediction [12]–[15].

Following the emergence of high-throughput experimental techniques that produce large amount of biological data, several studies have investigated robustness of a complex system in respect to the underlying network topology. Different types of networks have been studied with this purpose, such as metabolic networks [16], protein-protein interaction networks [17], [18] and regulatory interaction networks [19]. However, to our knowledge, robustness of network-based disease-gene prioritization methods, where the underlying network itself is perturbed, has not been extensively investigated. Due to the fact that network-based disease-gene prioritization methods use the connectivity between genes associated with the disease, we hypothesize that they may serve to distinguish diseases with respect to the predictability of causative genes via examining the behavior of network-based prioritization under noisy network models. The definition of robustness is problem specific [20] and here we define the robustness as the observed change in the prediction capacity of the prioritization methods when perturbing the underlying network.

In this work, our main goal has been to test the quality and robustness of several network-based disease-gene prioritization methods against perturbations introduced either in the underlying network or to known disease-gene associations. Next, to look into the relationship between robustness, functional diversity and modularity, we have examined the capability of these methods to identify disease modules (groups of genes that are enriched with the functions relevant to the disease). Through the analysis of the prediction performance of the network-based prioritization method under *in silico* perturbations and adopting a jackknifing scheme, we have categorized various disease-phenotypes (pathophenotypes) in Online Mendelian Inheritance in Man (OMIM) database [21] based on the level of recovery of the hidden disease-gene associations. Our results suggest that hidden disease-gene associations in several pathophenotypes, particularly the ones with high prevalence such as breast cancer and diabetes, can be recovered easier than the rest of the compared pathophenotypes, even when the underlying network is substantially perturbed. We have found that this was independent of the number of initial genes associated with the disease and rather mediated by the diversity of functions deduced by them. These findings provide evidence on the role of functional diversity in defining robustness of biological systems.

## Results and Discussion

### Network-based prioritization is sensitive to known disease-gene associations

**Figure 1:**
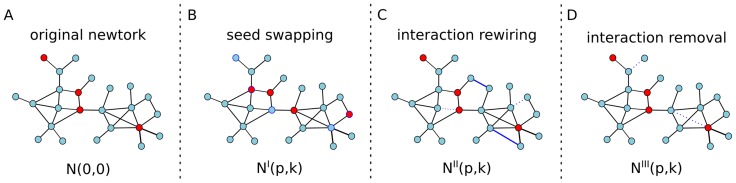
Different types of perturbations applied to the original network. (**A**) Original network, (**B**) seed swapping, (**C**) interaction rewiring, (**D**) interaction removal. *N(p,k)* represents the *k* ^th^ instance of the network with a perturbation level of *p* in a random ensemble of networks for a given perturbation type (see Methods).

Network-based disease gene prioritization methods rank the relevance of genes to a disease using known disease-gene associations (seeds) and the network topology. Any perturbation in the network topology or seeds induces a change on the ranking of the genes for the disease in concern. Systematically introducing perturbations at different levels and analyzing the changes in the ranking of the disease-genes provide a way to measure the robustness of the prioritization (see Figure 1 for an overview of the perturbations applied in this study). In order to assess the tolerance of a prioritization method to the noise in the underlying network or in the known disease-genes we used the jackknifing technique. This is a blind test in which we hid the disease-gene association of some seeds and we used the remaining seeds and a prioritization method to predict genes associated with the disease. Then, we checked whether the hidden seeds were predicted accurately by the prioritization. We tested five prioritization algorithms: NetShort, NetZcore and NetScore, three algorithms we have recently proposed that use global network topology [15]; and two existing algorithms, Functional Flow [22] and PageRank with priors [11]. The human protein-protein interaction (PPI) network (referred simply as network hereafter, unless otherwise stated) was obtained by integrating protein-protein interactions from several publicly available repositories. The network consisted of 11250 nodes (gene products) and 59220 edges (physical interactions) connecting them. We analyzed 19 disease phenotypes that were compiled among the human disorders in OMIM [21]. The phenotypes were created by merging disorders in OMIM using keywords describing the phenotype (see Methods and Table S1).

**Figure 2:**
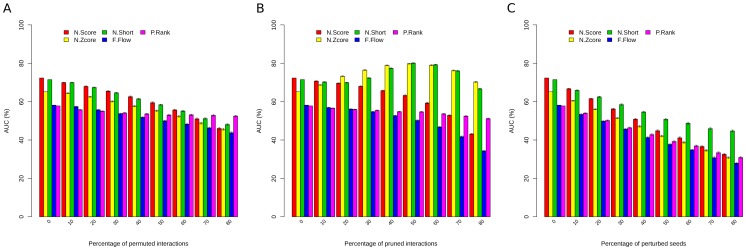
Robustness of the methods against the perturbation of the edges of the bPPI network and initial disease-gene associations. The interactions of the bPPI network were perturbed (**A**) by swapping the links in order to make false interactions or (**B**) by removing links. Plots show the average AUC and 95% confidence intervals calculated for the prediction of gene-disease associations for 19 diseases using NetScore (red), NetZcore (yellow), NetShort (green), Functional Flow (blue), and ToppGene (purple). The percentage of interactions swapped or removed varied between 0 and 80%. (**C**) Dependence on the number and quality of seeds. The average AUCs are given as the percentage of mis-annotated seeds goes from 0% to 80%.

We first questioned the robustness of the prioritization methods by means of testing the quality of the genes associated with a disorder. To address the dependence on the number of seeds, we replaced the seeds with non-seeds in the network at varying percentages (10% to 80%). That is, we disturbed the initial disease-gene associations at different levels by introducing wrong associations between genes and pathophenotypes. Then we calculated the area under Receiver Operating Characteristic (ROC) curve of network-based prioritization methods using a five-fold cross-validation setting on the perturbed disease-gene annotations. An increased percentage of mis-annotated seeds reduced the reliability of predictions for all methods (Figure 2a). If more than 70% of seeds were false, the area under ROC curve (AUC) reduced to less than 50% for all methods. Only NetShort resulted in an AUC higher than 50% when using 40% false seeds. In conclusion, all methods were dependent on the quality of the initial associations, but NetShort was less affected, compared to the rest, in predicting new genes associated with the disease. In order to ensure that this was not an artifact of the network, we repeated the analysis on the network used by Goh et al. [23]. This network contained a set of high confident protein-protein interactions in human (we refer to this network as Goh network hereafter, see Methods). The AUCs for the methods at different perturbation percentages using Goh network are given in Figure S1a and confirmed our observation.

### *In silico* analysis highlights the existence of alternative routes connecting disease-genes

To examine the effect of the quality of the interactions in prioritization, we randomly swapped the edges of the network. Also, to observe the relevance of the number of interactions, we randomly deleted edges of the network. The variation in the edges of the network ranged between 10% and 80%. We applied the prioritization methods to these perturbed networks and calculated the average AUC over all diseases as before. Figure 2b shows the decrease in AUC produced using false interactions (randomly swapped edges) for all methods. It is noteworthy that PageRank was the most robust method, the prediction performance of which was less affected from the perturbation of edges than the rest. On the other hand, edge deletion decreased the AUC for NetScore, Functional Flow and PageRank, but NetZcore and NetShort improved the prediction quality by increasing the AUC, and it only began to drop after more than 60% of the interactions were removed (Figure 2c). Repeating the same analysis with the Goh network revealed that the prioritization approaches exhibited a similar behavior (Figures S1b and S1c), indicating that these features were independent of the underlying network. Although this behavior was unexpected, it could be explained by the way the prioritization algorithms work [15]. These algorithms used the seed nodes to disseminate information through the network. For each disease, there were very few number of seeds compared to non-seeds in the network (varying in the range of 0.1–0.9% of all nodes, see Table S1). Therefore, random deletion of edges disconnected fewer seeds because the number of edges connecting a seed (either one seed with another seed or one seed with a non-seed) was much less than the number of edges connecting two non-seeds (only *<2%* of all edges involve a seed, see Table S2). Consequently, Functional Flow and NetScore were more affected than others because of their dependence on the number of paths that connected seeds with each other (Functional Flow simulates flow of information through links of the network and NetScore exploits multiple shortest paths connecting seeds). However, the effect of deletion diminished in the case of NetZcore since it normalized the score using random networks. Considering that disease-genes tend to be highly connected to each other, the scores of the nodes connected with seeds and thus the prioritization of the hidden disease-genes was improved. To understand this with an example, let's take a node *u* that is relatively more connected to seeds in comparison to any of the random networks. The random deletion of an edge would be more likely to remove a link connecting at least one non-seed. Hence, it would be more likely that node *u* would remain relatively more connected to seeds in comparison to random networks. NetShort, which accounted for the number of seeds involved in a path to identify shortest paths leading to seeds, also improved the quality of the predictions, due to the seeds being connected by alternative routes unaffected by the deletion of links (see Table S3 for the number of all shortest paths between pairs of seeds in each pathophenotype). Such backup circuits constitute a fail-safe mechanism and explain the resilient nature of cells [4].

**Figure 3:**
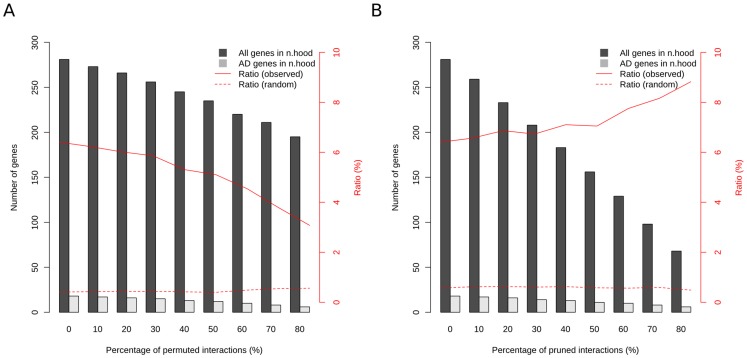
Change in the number and ratio of AD-related genes in the neighborhood of AD-seeds with respect to the amount of interaction permutation. The interactions of bPPI network were perturbed (**A**) by swapping the links in order to make false interactions or (**B**) by removing interactions. The percentage of interactions swapped or removed varied between 0 and 80%. The bars correspond to the average number of genes over perturbed networks (except the first bar in which the number of genes in the original network are given) whereas the line shows the ratio of the number of AD-related genes in the neighborhood of AD-seeds to the number of all genes in the neighborhood in the perturbed networks.

In order to gain an insight on the consequences of the changes in the network and explain the behavior of the prioritization algorithms independent of the jackknifing test described above, we focused on Alzheimer's Disease (AD), a relatively well studied pathophenotype for which we had an expert curated set of disease-gene associations [24]. We analyzed the connectedness of the genes associated with the AD in the perturbed networks introduced above. We used the genes associated with AD in OMIM [21] as seeds (AD-seeds). We took the neighbors of seeds in the network and checked how many of their neighbors are implicated in AD using an independent set of genes taken from literature (AD-related genes) [24]. We repeated this procedure on perturbed networks, interactions of which were either randomly swapped (permuted) or deleted (pruned) at different percentages (see Methods). Figure 3 shows the total number of genes and AD-related genes in the neighborhood of seeds. Not surprisingly, as the percentage of perturbation increased, the number of AD-related genes in the neighborhood of AD-seeds decreased. However, in the case of interaction pruning, the ratio of AD-related genes versus the total number of genes in the neighborhood of AD-seeds increased. This suggested that AD-related genes tended to remain connected with at least one AD-seed in the network. To confirm that this behavior was arising from the AD phenotype and not an artifact of the perturbations in the network, we checked the expected number of genes that would be covered at each perturbation level using 100 random sets of genes with sizes equal to the set of AD-related genes. The ratio of random gene sets covered in the neighborhood was substantially lower compared to the observed ratio of coverage of AD-related genes (Figure 3).

### Discovery of disease-genes under noisy network models varies among pathophenotypes

The analysis of the prediction performance caused by perturbations showed that disease-gene associations could still be discovered using network-based prioritization methods even when half of the interactions were perturbed (see Figures 2b and 2c). We questioned whether the emergence of such network-centric robustness of the prioritization methods depended on the pathophenotype. For this purpose, we defined, “prioritization tolerance” of a disease as the amount of perturbations required to cause a “critical” AUC change when we applied a network-based prioritization approach to predict its associated genes (see Methods for details). Among the different approaches to prioritize candidate genes we used NetScore for this analysis, since it had good overall prediction accuracy (Figure 2) and produced the smallest number of clusters coherently enriched in the functions associated with the corresponding disease (see Supplementary Results in Text S1 and Figure S2). Note that NetZcore had also similar properties, however the perturbation analysis above shoved that NetZcore was robust against perturbations whereas the AUC for NetScore fell down linearly as the percentage of perturbations increased (Figure 2). In order to minimize the bias on the prediction performance due to using a robust prioritization method and focus on the network-centric tolerance of the disease, we selected NetScore over NetZcore.

We grouped diseases into two categories based on the differences of the prioritization performance (assessed by the AUC) between the original network and perturbed networks using NetScore. For each pathophenotype, we checked the amount of perturbation (both for edge swapping and removal) required in the network that caused the prioritization performance fall below the critical AUC (table 1). If after perturbing more than 50% of the interactions, the prioritization of a disease still achieved a performance higher than the critical AUC, we labeled this disease as bearing high tolerance (referred as “tolerant” hereafter) in prioritization. On the other hand, if the critical AUC was reached with perturbations affecting less than 50% of the interactions in the network, we labeled the disease as bearing low tolerance (referred as “non-tolerant” hereafter). See Figure S3 for the changes in the NetScore prioritization AUC upon perturbations in the network for tolerant and non-tolerant diseases. According to our criteria, pathophenotypes such as *breast cancer, cardiomyopathy, diabetes, leukemia, obesity* and *parkinson disease* were tolerant and thus higher capability of adaptation against perturbations in the underlying network than the rest of the pathophenotypes. Tolerant pathophenotypes have been extensively studied and might have a larger number of known disease-gene associations than the rest. Therefore, it may be argued that this is the cause to be classified as tolerant diseases under our criterion. However, in the following section we show that this is not true.

**Table 1:** Network-based prioritization performance on the original and perturbed networks*.

Pathophenotype	AUC (%)	Pathophenotype	AUC (%)
	org.	crit.	perm.	del.		org.	crit.	perm.	del.
alzheimer	78.3	64.2	62.5	62.8	lung cancer	85.0	67.5	65.8	68.4
anemia	70.3	60.2	56.4	57.9	lymphoma	79.7	64.9	62.3	71.8
ataxia	62.6	56.3	54.2	53.8	mental retardation	56.3	53.2	46.6	45.3
**breast cancer**	76.7	63.4	70.7	75.8	myopathy	86.0	68.0	67.3	72.0
**cardiomyopathy**	69.5	59.8	65.0	70.5	**obesity**	72.0	61.0	67.4	70.4
cataract	72.0	61.0	53.9	52.8	**parkinson disease**	80.0	65.0	70.9	78.5
**diabetes**	61.4	55.7	58.4	63.4	prostate cancer	68.0	59.0	52.7	62.7
epilepsy	62.1	56.1	47.4	47.4	schizophrenia	53.3	51.7	40.9	42.1
hypertension	70.0	60.0	47.7	51.8	systemic lupus erythematosus	86.3	68.2	64.2	72.7
**leukemia**	84.6	67.3	75.8	81.6					

*Table shows the AUC values using the original network (org.), the critical AUC values (crit.) and the AUC values using perturbed networks (perm.; 50% of interactions permuted, del.; 50% of interactions deleted) for each pathophenotype. Tolerant pathophenotypes are highlighted with bold case.

### Comparison between pathophenotypes implies a role for functional diversity in determining prioritization tolerance

**Figure 4:**
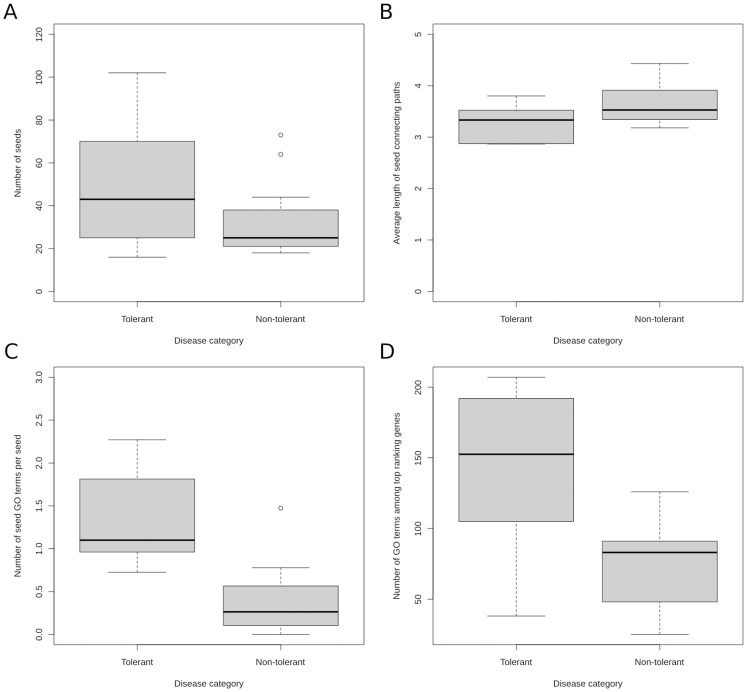
Comparison of tolerant and non-tolerant pathophenotypes. With respect to (**A**) known disease annotations associated with disease, (**B**) the connectivity of these seeds (assessed by the average shortest path length between seeds), (**C**) the ratio of the number of seed GO terms to the number of seeds associated with the diseases, and (**D**) the number of GO terms enriched within the high scoring subnetwork of the diseases.

We checked whether the diseases in different categories bore similar properties: 1) in terms of known disease annotations associated with disease (seeds); 2) in terms of connectivity of these seeds; and 3) in terms of functions enriched among these seeds. In principle, it is reasonable to think that the prioritization tolerance might depend on the number of seeds of the phenotype. This would also suggest an explanation to the fact that diseases of high prevalence were categorized as tolerant, simply because they were more studied. Tolerant diseases had slightly higher number of seeds, nevertheless, there was not a significant difference between the number of seeds of tolerant and non-tolerant diseases (Figure 4a, associated p-value with the two-sided Wilcoxon rank sum test, *p = 0.27*). In order to make sure that number of seeds has no significant effect on the categorization, we took the diseases with less than 50 seeds in each category and confirmed that tolerant diseases (with less than 50 seeds) still had higher AUC values on average compared to non-tolerant diseases (with less than 50 seeds) upon perturbations (one-sided p-values were 5.5e-3 and 0.04 for interaction swapping and deletion respectively). In our previous study [15], we had shown that the prediction performance of network-based disease-gene prioritization methods was inversely correlated with the average shortest path length between seeds of a phenotype. Nevertheless, seeds of tolerant pathophenotypes were not distinguished significantly from the rest with respect to the average length of shortest paths between seeds (Figure 4b, *p* *= 0.13*). We have to note that the average length of shortest paths connecting two seeds is independent of the number of seeds. We also investigated the difference between the number of shortest paths connecting seeds (normalized by the number of seeds per pathophenotype) among two groups of diseases and found not significant discrepancy (*p = 0.15*). No significant difference between the number of interaction partners (degrees) of the seeds of tolerant and non-tolerant diseases were observed either (*p = 0.24*).

Next, we looked at the functional enrichment of seeds involved in the two groups of diseases. We calculated the enrichment of GO terms of seeds (i.e., seed GO terms, see Methods for details) for both categories. In order to avoid possible bias towards the number of seeds, we normalized the number of seed GO terms dividing them by the total number of seeds for each pathophenotype. Considering that tolerant diseases had slightly more number of seeds (as mentioned above), normalizing by total number of seeds ensures that the observed number of GO terms are penalized more for tolerant diseases. Still, pathophenotypes with high prioritization tolerance contained higher ratio of seed GO terms per seed (around 3 times more than that of non-tolerant pathophenotypes), proving that a larger number of biological functions associated with seeds were involved in tolerant diseases than in pathophenotypes with low prioritization tolerance (Figure 4c, *p* *= 3.29e-3*). Likewise, on average there were almost twice as many GO terms enriched within the high scoring subnetworks (top 5% scored nodes in the network using NetScore) of tolerant diseases compared to non-tolerant diseases (Figure 4d, *p* *= 0.03*). These findings suggested that tolerant pathophenotypes tended to be functionally more diverse, being involved in a larger number of functions compared to non-tolerant phenotypes. Further analysis on the GO terms shared between pathophenotypes suggested that tolerant diseases tended to share more GO terms among themselves than non-tolerant diseases (Figure S4 and Figure S5).

### Differential network analysis reveals a connected core machinery in breast cancer

**Figure 5:**
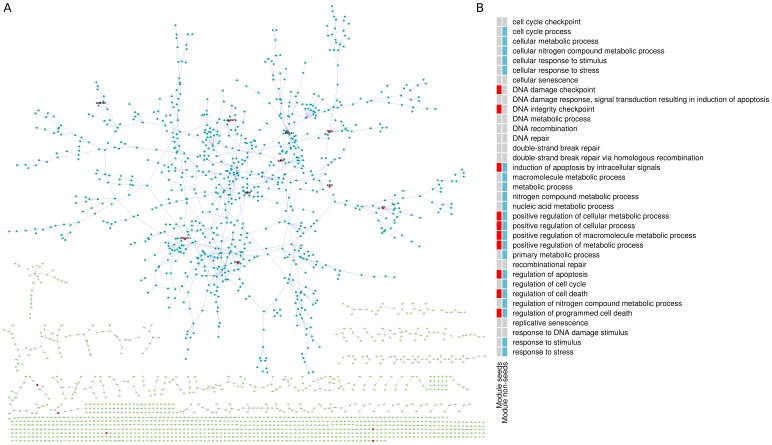
Differential network of protein-protein interactions for breast-cancer. (**A**) The network containing interactions common to the two pruned networks with which the NetScore obtained the best prediction performance for breast cancer but did not exist among the common interactions in the worst performing two pruned networks. The red nodes are seeds. (**B**) The seed GO terms that are covered in the largest connected component of this differential network via using only seed genes (red) and only non-seed genes (blue) in the component.

Breast cancer was one of the pathophenotypes that was observed to bear high prioritization tolerance upon the analysis of network-based prioritization on the ensemble of randomly perturbed networks. To gain insights on the interactions giving rise to network-centric tolerance in breast cancer, we focused on the perturbed networks in which 80% of the interactions were randomly removed and chose among the random networks those with highest and lowest prediction accuracy when the network-based prioritization method was applied. We selected two networks in each case, two networks with AUCs of 82% and 73.9% and two networks with AUCs of 46.1% and 46.9%. Then, we checked the set of interactions yielding the difference between highest and lowest AUC. For this, we considered the interactions that were common in the two networks with highest AUC but were not among the common interactions of the two networks with lowest AUC. These interactions potentially caused that breast cancer phenotype was classified as tolerant. The network consisting of these interactions was named differential network (see Figure 5a). One key observation in this network was that the largest connected component (referred as module hereafter) of the differential network contained one third of the seed genes and this included proteins encoded by BRCA1, TP53, ESR1, AR, AKT1. This is an interesting result, because the networks from which the differential network was obtained contained only one out of five of the original interactions. Moreover, the GO terms enriched in this module covered 65.7% of all 35 seed GO terms of breast cancer (see Tables S4 and S5). Next, we checked the GO terms enriched using only non-seeds in this module. Among 312 GO terms enriched with these genes, 22 were seed GO terms yielding a highly significant enrichment of seed GO terms (*p = 5.97e-7*). This confirmed that the enrichment was not originated solely from the 10 seeds of the component. Figure 5b shows the seed GO terms covered by seed and non-seed genes of this module. We concluded that the observed prioritization tolerance was due to i) redundancy, being difficult to disconnect seed proteins with the random deletion of interactions and ii) functional diversity, the existence of a large connected module involving genes with the characteristic functions of the disease.

### Implications of this study and future directions

In this study, we have analyzed the robustness of several disease-gene prioritization algorithms using known gene-disease associations and protein-protein interaction networks. Our analysis on randomly perturbed interaction networks pointed to the existence of backup circuits within the PPI network constituting a fail-safe mechanism. Strikingly, the performance of the methods might tolerate up to 50% of interactions being removed, the point at which methods using alternative paths start suffering from low prediction performance. NetZcore and NetShort showed to be consistently effective in ranking genes when interactions were removed, while PageRank was more robust against the introduction of false interactions (we have to note that though it was more robust than NetShort, its overall performance was worse). Furthermore, NetShort, the method that calculated the distance to another gene in the network taking into account number of known disease-genes included in the path, yielded better prioritization performance when the known disease-gene information was noisy.

We categorized diseases with respect to their prioritization tolerance based on the performance of a network-based prioritization method under the perturbation of the network. Interestingly, pathophenotypes with high prioritization tolerance included many diseases with high prevalence in society. We further investigated whether there were characteristic differences between diseases involved in these categories. Neither the number of seeds, nor the average length of the shortest paths between them were significantly different between tolerant and non-tolerant diseases. On the other hand, the number of GO-terms enriched among seed genes (known disease-genes) per seed stood out as the most important factor in defining prioritization tolerance of a pathphenotype. That is, a disease was more likely to be tolerant to noise in the interaction network if the genes involved in that pathophenotype were functionally more diverse. Futhermore, tolerant diseases showed a higher tendency to share functions with other pathophenotypes. Such common features at the functional level might be useful for understanding the etiology of complex genetic disorders [25]. We have presented a way of systematically assessing the prioritization tolerance of pathophenotypes emerging from the underlying protein-interaction network. Although the robustness of a disease cannot possibly be explained only by the topology of the network, intuitively the prioritization tolerance of a disease could imply its robustness. Previous studies have argued that cancer [26] and diabetes [27] are robust diseases through a holistic analysis pointing out the redundancy and feedback mechanisms in these diseases. These studies are in agreement with our results on several diseases with high prioritization tolerance such as breast cancer, diabetes and leukemia. Furthermore, in addition to functional redundancy and decoupling, two key features mediating robustness of biological systems [26], [28], functional diversity (i.e. involving multiple pathways) may be related to the robustness of diseases. On the other hand, we did not find lung and prostate cancers as bearing high prioritization tolerance. Considering the heterogeneity among different cancer types, it is not very surprising to see such differences with respect to prioritization tolerance for various types of cancer. In fact, even within a certain type of cancer, such as breast cancer, a certain level of heterogeneity is expected at the patient level. In this sense, the genes involved in the pathophenotype and the underlying PPI network might be significantly different compared to another patient [29]–[31]. Therefore, the major limitations of our study are the incompleteness and inaccuracy of the disease-gene associations and protein-protein interactions. Nonetheless, the developments in high throughput genomics and proteomics screening, as well as experimental and computational efforts in identifying context-specific interaction networks (e.g., tissue-specific) [32], [33], will help to build more comprehensive models explaining the plasticity of diseases.

The findings presented here may help developing novel network-medicine approaches that try to characterize the interconnected pathways implicated in diseases and possibly suggest points of action to compensate the changes induced by the disease [34]. Functional diversity can explain why polypharmacological approaches, which typically target many gene products simultaneously via administration of multiple drugs [35], may work better on some diseases such as cancer [36]–[38].

## Methods

### Gene–phenotype associations

Genes and their associated disorders were taken from Online Mendelian Inheritance in Man (OMIM) database [21]. OMIM is one of the most comprehensive and reliable repositories of genes with Mendelian mutations and the disorders associated with them. Phenotypic associations for genes were extracted from the OMIM Morbid Map (ftp://ftp.ncbi.nih.gov/repository/OMIM/morbidmap retrieved August 27, 2009) by searching for keyword entries associated with the disorders given in Table S1. A disorder was considered if at least 25 genes were associated with it in the Morbid Map after merging several diseases under the same keyword (e.g., for Alzheimer's disease we collected Alzheimer's disease types 1, 2, etc. using the keyword “alzheimer”). Table S1 summarizes all diseases used under the context of this study, the number of genes associated with them and number of protein products these genes encode in the PPI network. Asthma, neuropathy and spastic paraplegia also satisfied the criterion of having at least 25 genes, however they were excluded from the robustness analysis since the AUCs using the original network were lower than 50% (see below).

Genes associated with a disorder were mapped to their products (proteins) in the PPI network and assigned an initial score for their phenotypic relevance. Thus, proteins translated by genes known to be involved in a particular pathology were termed *seeds* and have the highest score (*1.0*) in the network. All other proteins in the network were assigned a *non-seed* score (lowest score in the network: *0.01*). The correspondence between genes and their products (proteins) was determined using the data integration protocol of BIANA [39].

### Protein–protein interaction network

We used the human PPI network presented in our recent work [15]. The interaction network was compiled from publicly available major interaction data repositories using BIANA integration tool (Table S6). First, protein-protein interactions from different sources were integrated with BIANA [39] to obtain a human interactome. High throughput pull down interaction detection methods introduce many indirect relationships (such as being involved in the same complex) in addition to direct physical interactions. Thus, we removed the subset of interactions obtained by tandem affinity purification and called this network as the bPPI (binary protein-protein interaction) network. We also used the human interactome from Goh et al. [23] (referred to as the Goh network), which combined two high quality yeast two-hybrid experiments [40], [41] and protein-protein interactions obtained from the literature.

### Network-based prioritization of disease-genes

To assess the tolerance of a given phenotype to the noise in the underlying network or in the seeds, we used five network-based prioritization algorithms available in *GUILD* software package. *GUILD* (Genes Underlying Inheritance Linked Disorders) is a network-based disease gene prioritization framework [15], [42]. The prioritization methods rank the nodes of the network according to their implication in the pathopheontype. The network-based prioritization approaches obtain this rank by disseminating the information of seeds through the network. *GUILD* framework provides several methods that use known disease-genes and their interactions to rank the relevance of genes in a disease or disorder. The basic hypothesis is that genes whose proteins interact with each other tend to exhibit similar features, such as function and/or phenotype. These methods require an initial set of genes associated with a particular phenotype (e.g., a Mendelian disorder) and interactions between the products of these genes. We chose three topology-based ranking algorithms: NetShort, NetZcore, and NetScore (see Supplementary Methods in Text S1 for details); and two state-of-the-art algorithms PageRank with priors [43] (as used in ToppNet [11]) and Functional Flow [22]. PageRank with priors has recently been proven its success in network-based disease-gene prioritization [11], [12], [14].

To evaluate the prioritization methods, we used five-fold cross validation and calculated area under ROC curve (AUC). The AUC*s* were averaged over all folds. The details of the evaluation has been described elsewhere [15].

### Dependency of prioritization methods on network features and gene associations

We evaluated the dependencies of the methods by modifying the input data using three tests: 1) permuting interactions at random, 2) randomly removing interactions of the network, and 3) permuting the seeds at random. We tested the effect of the modifications on the bPPI and Goh networks using OMIM gene-phenotype associations. The degree of modifications ranged from 10% to 80% for each network and seed set. Ensembles of 100 random networks and random seed-sets were used to assess average prediction performance for each perturbation level. For the first test, nine groups of 100 networks were generated by swapping the edges of the original network (randomly creating new edges and removing old ones), and each group contained a different number of random permutations corresponding to the 10% to 80% of the number of edges. For the second test, the edges were randomly deleted to create nine groups of 100 networks in which the number of deletions varied between 10% and 80% of the number of edges. For the third test, a varying percentage of seed nodes (10% to 80%) was replaced with non-seed nodes 100 times, yielding nine groups of seed-sets and the percentage of non-seeds in each group ranged between 10 and 80. The prioritization methods were applied to these modified data sets and for each group, the AUC was averaged over the 100 randomly modified networks or seed sets. That is, for each level of perturbation *p* (%), the average AUC over an ensemble of randomly perturbed networks of size n can be written as follows;
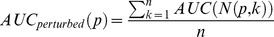
where *N(p,k)* is *k* ^th^ instance of the network with a perturbation level of *p* in a random ensemble of networks for a given perturbation type

### Functional enrichment analysis

GO terms enriched among genes corresponding to high scoring proteins in a network (top 5% nodes in the network with respect to their prioritization score) were identified using the FuncAssociate2.0 [44] web service. Proteins in the network were mapped to the genes using the gene symbols provided by UniProt, and these symbols were fed to the web service. All genes in the network were used as the background gene list. A GO term was associated with the gene set, if and only if, the adjusted p-value associated with the term was less or equal to *0.05*. Similarly, seed GO terms of pathophenotypes (or disease GO terms) were defined as the GO terms enriched among the seeds used in the prioritization method for that disease. For the comparison analysis of tolerant and non-tolerant pathophenotypes, the semantically non-redundant GO terms that belonged to biological process ontology were taken into consideration. GoSemSim R package [45] used to calculate the semantic similarity based on the metric proposed by Wang et al. [46] and semantically non-redundant GO terms were identified by removing the term that had at least 90% similarity to another term and that was closer to the root of the ontology (in case of a tie, one of the two terms was taken randomly). In the differential network analysis of breast cancer, a p-value was calculated assuming a hypergeometric distribution and using 1320 GO biological process terms enriched among all the genes in the original network as the set of all possible GO terms (all human genes were used as background in this case).

### Defining network-centric tolerance of a pathophenotype

For the analysis of network-centric tolerance, we used the NetScore method of the GUILD package since *1*) it had the highest prediction performance, *2*) it produced clusters that are functionally more relevant to the disease compared to the rest of the prioritization methods, and *3*) the method itself was not robust against perturbations (Figure 2 and Figure S2). We defined network-centric tolerance based on the amount of interaction perturbation required to cause a “critical AUC change” in network-based prioritization of a disease. For each disease, the “critical AUC change” was set as half of the AUC difference and the expected AUC that would be obtained by random predictions (i.e. 0.5). That is, for each pathophenotype, the “critical AUC change” was calculated using the following formula:
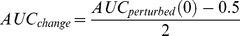
and accordingly the “critical AUC” of a pathophenotype was given by:
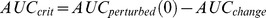

Figure S6 gives a schematic explanation of the critical AUC change. A disease phenotype was called tolerant if the amount of interaction perturbation (both interaction swapping and removal) required to cause a “critical AUC change” was lower than 50%. Similarly we called a disease phenotype non-tolerant if the amount of interaction perturbation for “critical AUC change” was higher than 50%. This can be formulated as follows:
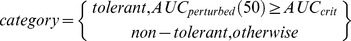
For testing the significance of differences in the distribution of values between robust and non-robust diseases we used Wilcoxon rank-sum test. Alpha values were set to 0.05. R software (http://www.r-project.org) was used to compute the statistics.

## Supporting Information

Figure S1 **Robustness of the methods against the perturbation of the edges of the Goh network.** Plot of the average AUC (shown in bars) and confidence interval (shown with error lines) calculated for the prediction of gene-disease associations by NetScore (red), NetZcore (yellow), NetShort (green), Functional Flow (blue), and PageRank (purple). The interactions of Goh network were perturbed (**A**) by swapping the links in order to make false interactions or (**B**) by removing interactions. The percentage of interactions swapped or removed varied between 0 and 80%. (**C**) Plot of the average AUC and confidence intervals calculated for the prediction of gene-disease associations as the percentage of mis-annotated seeds goes from 0% to 80%.
(EPS)

Figure S2 **Module-based functional enrichment analysis of prioritized subnetworks.** (**A**) Number of modules identified in the neighborhood of known disease associations (N.hood) and in high scoring subnetworks identified by NetScore (N.Score), NetZcore (N.Zcore), NetShort (N.Short), Functional Flow (F.Flow) or PageRank with priors (P.Rank) prioritization methods. (**B**) Percentage of seed GO terms (GO terms significantly enriched in the set of genes associated with the disease) among all GO terms significantly enriched in the identified modules.
(EPS)

Figure S3 **AUC change at different levels of perturbations in the network.** (**A**) Tolerant and (**B**) non-tolerant pathophenotypes. Solid lines correspond to AUC of the disease when NetScore prioritization method is applied to the interaction network whose edges are randomly swapped (averaged over all such networks) and dashes correspond to AUC of the disease when the prioritization method is applied to the interaction network whose edges are randomly deleted.
(EPS)

Figure S4 **Heatmap representation of Jaccard indices calculated for every pair of pathophenotypes.** We used GO terms in the high scoring (top 5%) modules identified by NetScore prioritization method. Tolerant pathophenotypes are on the top of the heatmap (green), whereas non-tolerant pathophenotypes are at the bottom (orange). No overlap is represented with gray color and the degree of overlap increases as color gets darker (from white to blue).
(EPS)

Figure S5 **GO terms shared in the high scoring modules of at least 4 diseases.** Tolerant pathophenotypes are on the left of the heatmap (green) and non-tolerant pathophenotypes are on the left (orange).
(EPS)

Figure S6 **Schematic explanation of the critical AUC change.**
(EPS)

Table S1 **The number of genes associated with the diseases used in this study and the number of gene products corresponding to these genes in the interaction networks.**
(XLS)

Table S2 **The number of seed connecting and non-seed connecting edges in the interaction networks for each disease.**
(XLS)

Table S3 **The number of shortest paths connecting pairs of seeds in each disease.**
(XLS)

Table S4 **GO term enrichment of the genes in the largest connected component of the differential network for breast cancer.**
(XLS)

Table S5 **Seed GO terms (GO terms enriched by the seed genes) for all diseases.**
(XLS)

Table S6 **The interaction sources used to create the human protein-protein interaction network.**
(XLS)

Text S1 (DOC)
