# Supplementary material for: Analysis of the Robustness of Network-Based Disease-Gene Prioritization Methods Reveals Redundancy in the Human Interactome and Functional Diversity of Disease-Genes
Source: PLoS One. 2014 Apr 14;9(4):e94686. doi: 10.1371/journal.pone.0094686 (PMC3986215; doi:10.1371/journal.pone.0094686)
Supplement: Text S1 — (DOC) [file pone.0094686.s013.doc]

*Supplementary material for Guney E. and Oliva B., Analysis of the Robustness of Network-based Disease-gene Prioritization* *Methods**Reveals Redundancy in the Human Interactome and Functional Diversity of Disease-genes*

**SUPPLEMENTARY RESULTS**

*Investigating functional modularity of the top-ranking genes identified by the prioritization methods and selection of prioritization method for prioritization tolerance analysis*

Cells exhibit a modular nature in which genes are organized into various interconnected pathways and such organization of genes is often attributed as an essential component of robustness [1]. We questioned whether the prioritization methods identified groups of genes that were functionally associated with the disease phenotype. First, we carried out a module-based functional enrichment analysis, using top scored nodes (the highest scoring 5% of the all nodes in the network) associated with a disease identified by network-based prioritization approaches. Then, we examined the extent to which these prioritization algorithms were able to discover functions implicated in the disease. We acquired GO terms that are significantly enriched in the set of genes associated with the disease (seed GO terms). We used five network-based prioritization approaches to score genes associated with diseases from the OMIM database. We also used a trivial prioritization approach where all neighbors of the seeds were prioritized for the same pathophenotype (direct neighborhood). Next, we applied a clustering protocol based on MCL [2], a random-walk based graph clustering algorithm, to the sets of highly scored nodes or the neighbors of the seeds. For each disease, this procedure produced a set of clusters of proteins potentially implicated in the pathology of the disease. A cluster was potentially implicated in the disease, if and only if it contained two or more seeds (see Supplementary Methods for details). These clusters of highly ranked proteins in the network can be thought as disease modules and can be used to study the implication of several biological processes in various diseases. We checked the percentage of seed GO terms among all GO terms significantly enriched in these clusters, based on the GO terms enriched among the genes in the clusters. The number of clusters was smaller when prioritizing with NetScore and NetZcore than with the rest of methods (Figure S2a). Moreover, the genes in the clusters prioritized by NetScore and NetZcore were slightly more enriched in seed GO terms, suggesting that they could identify genes more relevant to the disease, these being either seeds or genes involved in similar functions (Figure S2b). In particular, the GO term enrichment in the clusters when prioritizing with the neighborhood approach was very low, while the number of clusters was large. We wish to note that if the number of clusters is large, the seeds may be functionally spread among several sets. In contrast, if the number of clusters is small, the genes (or proteins) in these clusters have similar functions that may characterize the main functional features of the disease. Our results showed that NetScore and NetZcore tended to form a small set of functionally coherent groups identifying groups of genes with functions similar to the known disease-genes. Since NetZcore was more robust than NetScore against perturbations, NetScore was chosen for further analysis of prioritization tolerance of pathophenotypes.

We also calculated the Jaccard index for quantifying GO terms shared between each pair of diseases enriched in the top ranking genes (see Figure S4). Tolerant diseases showed a higher tendency to share functions among themselves than non-tolerant diseases. A list of GO biological processes common to at least three diseases are given in Figure S5. There were few GO terms shared by the diseases supporting the contribution of the abundance of the functions in prioritization tolerance of the diseases rather than specific individual functions.

**SUPPLEMENTARY METHODS**

**Network-based disease-gene prioritization methods**

We recently proposed three disease-gene prioritization methods that use known disease-gene annotations and protein-protein interaction network to rank genes (or their proteins) with respect to a given phenotype [3,4]. The first method, named *NetShort,* considers not only the number of links that reach to the phenotype-associated node but also the number of phenoype-associated nodes that are included in the path. This is achieved by modifying the weight of the links in shortest path algorithm such that the links connecting seed nodes are shorter than the links connecting non-seed nodes. The second method, *NetZcore,* iteratively assesses the relevance of a node for a given phenotype by averaging the normalized scores of the neighboring nodes in a network. The normalized scores for each node is calculated using observed scores of the same node in a catalog of random networks that have the same topology as the original network. Finally, the third method, *NetScore* considers multiple shortest paths (if exist) from the source of information to the target for each node and ignores all other paths between them. All of these algorithms were implemented in C++ (requires GNU C++ compiler and GNU make) and freely available at <http://sbi.imim.es/GUILD.php> .

**Identification of clusters associated with the disease.** We defined two types of subnetworks to compare the enrichment of GO terms and specific functions in putative pathways: 1) a trivial approach also named “neighboring” used the nodes of a network formed by seeds of a disease and their interaction partners; and 2) a more sophisticated approach, using the highly scored nodes (proteins) of the network identified by a prioritization method. The edges of these subnetworks were taken from the original network, using all edges that connected any of the selected nodes. For assigning GO terms we used the genes that produce these selected proteins. We identified clusters in these subnetworks using the Markov cluster (MCL) algorithm [2]. The inflation parameter of MCL was chosen to be *1.7*, the optimum value obtained by Brohee and van Helden [5]. Only the clusters that contain two or more proteins translated by disease-associated genes were taken into consideration. We then determined the percentage of GO terms significantly enriched in the set of genes associated with the disease (seed GO terms) among all GO terms significantly enriched in identified clusters.

**SUPPLEMENTARY REFERENCES**

1. Kitano H (2004) Biological robustness. Nat Rev Genet 5: 826–837.

2. Enright AJ, Van Dongen S, Ouzounis CA (2002) An efficient algorithm for large-scale detection of protein families. Nucleic Acids Res 30: 1575–1584.

3. Guney E, Oliva B (2011) Toward PWAS: discovering pathways associated with human disorders. BMC Bioinformatics 12: A12. doi:10.1186/1471-2105-12-S11-A12.

4. Guney E, Oliva B (2012) Exploiting Protein-Protein Interaction Networks for Genome-Wide Disease-Gene Prioritization. PLoS ONE 7: e43557. doi:10.1371/journal.pone.0043557.

5. Brohee S, van Helden J (2006) Evaluation of clustering algorithms for protein-protein interaction networks. BMC Bioinformatics 7: 488.
